# Ultrasound-Assisted Extraction of Polyphenols from *Hericium erinaceus*: Optimization, Bioactivities and LC-MS-Based Chemical Profiling

**DOI:** 10.3390/molecules31071138

**Published:** 2026-03-30

**Authors:** Hongfei Liu, Cong Zhao, Shuyue Pang, Yuting Shu, Lina Chen, Jing Wang, Helong Bai

**Affiliations:** 1 College of Chemistry, Changchun Normal University, Changchun 130032, China; qx202308010@stu.ccsfu.edu.cn (H.L.); qx202308024@stu.ccsfu.edu.cn (S.P.); qx202408003@stu.ccsfu.edu.cn (Y.S.); chenlina4321@163.com (L.C.); 2 Institute of Science and Technology Innovation, Changchun Normal University, Changchun 130032, China; 3 College of Life Sciences, Changchun Normal University, Changchun 130032, China; zhaocong@ccsfu.edu.cn

**Keywords:** *Hericium erinaceus*, extraction optimization, antioxidant activity, α-amylase, α-glucosidase

## Abstract

In this study, the Box–Behnken Design (BBD) was adopted to optimize the ultrasound-assisted extraction (UAE) conditions of polyphenols from *Hericium erinaceus* (*H. erinaceus*) on the basis of single-factor experiments, with extraction time, solid–liquid ratio and ethanol concentration as the key investigation factors. The optimal extraction parameters were determined as follows: extraction time of 56.85 min, solid–liquid ratio of 1:56.71 g/mL and ethanol concentration of 44.64%, under which the actual yield of the total polyphenol crude extract (TPCE) reached 0.9985 ± 0.03%, which was highly consistent with the theoretical predicted value of 0.9960%, verifying the good fitting degree of the established model. Taking L-ascorbic acid as the positive control, the antioxidant activity of TPCE was evaluated by determining its scavenging capacity against ABTS·^+^, ·OH and DPPH· free radicals, and the half-maximal effective concentration (*EC*_50_) values were measured to be 0.8850, 0.9490 and 4.198 mg/mL, respectively. With acarbose as the reference drug, the inhibitory effects of TPCE on α-amylase and α-glucosidase related to carbohydrate metabolism were assayed, and the corresponding half-maximal inhibitory concentration (*IC*_50_) values were 0.0135 and 130.3 mg/mL, respectively. Furthermore, ultra-high performance liquid chromatography-quadrupole time-of-flight mass spectrometry (UHPLC-QTOF-MS) was employed for the tentative identification of bioactive components in TPCE, and a total of 48 and 64 chemical constituents were characterized in negative and positive ion modes, respectively, providing a chemical basis for the biological activities of TPCE. This study confirmed that UAE is an efficient and feasible technology for extracting polyphenols from *H. erinaceus*, which lays a theoretical foundation for the development and utilization of its polyphenols, and also provides novel insights into the development of natural functional ingredients and potential therapeutic agents for the intervention of type 2 diabetes. Additionally, the findings further validate edible fungi as a valuable reservoir of natural bioactive substances, with promising application prospects in the research and development of functional foods and pharmaceuticals targeting metabolic diseases.

## 1. Introduction

Edible mushrooms are widely appreciated for their nutritional properties, as well as for their biological activities and therapeutic potential. Regarding medicinal values, mushrooms have emerged as an important source of compounds with antioxidant, antimicrobial and antitumor features [1]. *H. erinaceus* has historically been broadly cultivated and widely consumed as traditional medicinal herbs as well as functional food in the orient for several hundred years [2]. Members of the genus *H. erinaceus* produce fleshy, whitish basidiomata, such as *H. erinaceus* coralloides (Scop.) Pers. (in *Hericiaceae*, Russulales, Agaricomycetes, Basidiomycota) [3]. It is distributed across the Northern Hemisphere in Europe, Asia and North America [4]. There are 73 records in the Fungus Index (http://www.indexfungorum.org/Names/NAMES.ASP (accessed on 26 March 2026)), indicating that *H. erinaceus* is rich in a variety of functional active ingredients, with various physiological functions such as anticancer and enhancing immunity [5]. Studies have shown that, in addition to functional macromolecules such as polysaccharides and proteins, *H. erinaceus* also contains small molecular active components such as terpenoids, cerebrosides, phenols and sterols [4]. These bioactive compounds have various physiological activities in healthcare functions [6].

Plant polyphenols, secondary metabolites in plants, possess valuable functional properties, including anti-inflammatory, antioxidative and hypoglycemic effects [7]. These substances vary from simple molecules to complex structures, all featuring benzenic rings with two or more hydroxy groups. These active components are essential for development, fertility, and immunity against infections, pathogens, and diseases. Polyphenolic compounds are naturally occurring biologically active compounds that have been thoroughly researched due to their beneficial impact on health [8,9]. In addition to their therapeutic roles, polyphenols contribute to the taste, appearance, and organoleptic characteristics of foods [10]. A variety of compounds have been identified in *H. erinaceus* across different studies, including acetohydroxamic acid, catechin hydrate, resveratrol, myricetin, fumaric acid, gallic acid, protocatechuic acid, 4-hydroxybenzoic acid, phloridzin dihydrate, 2-hydroxycinnamic acid, naringenin, quercetin, and luteolin [11]; 3,4-dihydroxybenzoic acid, caffeic acid, syringic acid, rutin, ellagic acid, p-coumaric acid, salicylic acid, vanillin, ferulic acid, sinapic acid, rosmarinic acid, and t-cinnamic acid [12]; α-resorcylic acid and 4-coumaric acid [13]; as well as succinic acid, catechin, and 2-hydroxybenzoic acid [14]. Based on the above, it is necessary to conduct in-depth research on polyphenols from *H. erinaceus*.

The use of ultrasound-assisted extraction (UAE) of bioactive compounds has been in-creasing because it is a good alternative to the conventional extraction methods. UAE was used to maximize total polyphenol content [15,16]. Also, two primary systems have been widely employed in ultrasonic extraction: ultrasonic probes and ultrasonic baths. Previous studies have demonstrated that ultrasonic probes exhibit superior efficiency in extracting active components [17], while ultrasonic baths offer the advantage of protecting the structural integrity and biological activity of target active substances throughout the extraction process [18]. The selection of extraction solvent for ultrasonic-assisted extraction is also critically important. Although methanol has been reported to be the optimal solvent for extracting total phenolic compounds from *G. applanatum* and *F. fomentarius*, ethanol was still employed as the extraction solvent in this study. Distinct from the commonly used methanol, ethanol is classified as a GRAS (Generally Recognized as Safe) solvent, which constitutes the core reason for selecting ethanol as the extraction solvent in the present experiment [19,20].

Type 2 diabetes mellitus and its related complications are growing public health problems. Many natural products including polyphenols can be used in treating and managing type 2 diabetes mellitus and different diseases, owing to their numerous biological properties [21]. Controlling carbohydrate digestibility by inhibiting starch digestive enzyme (α-amylase and α-glucosidase) activities is an efficient strategy to control postprandial hyperglycemia [22]. Hyperglycemia in DM is known to increase reactive oxygen species production, enhancing oxidative stress and glycation, which in turn contributes to the development of advanced glycation end products, exacerbating diabetic complications [23]. The potent antioxidant capabilities of polyphenols can help battle oxidative stress, one of the key factors in the pathogenesis of diabetes. Although *H. erinaceus* contains phenolic components and exhibits diverse physiological activities, studies on its polyphenol extraction, antioxidant and enzyme inhibitory activities, as well as their links to hypoglycemic effects, remain limited and insufficient.

In order to fully explore the potential of *H. erinaceus* polyphenols as natural antioxidants and hypoglycemic active substances, and fill the research gap in this field, polyphenols yield was used as the core evaluation index in the present study to optimize the extraction process of polyphenols from *H. erinaceus*. On this basis, the antioxidant activity and starch digestive enzyme inhibition activity of the extracted polyphenol components were systematically investigated, aiming to provide experimental data and theoretical support for the in-depth development and utilization of *H. erinaceus* as a functional food resource for the prevention and auxiliary treatment of type 2 diabetes mellitus.

## 2. Results and Discussion

### 2.1. Optimization of Polyphenol Extraction Procedure

#### 2.1.1. Effect of Different Factors on Polyphenol Yield

**Figure 1:**
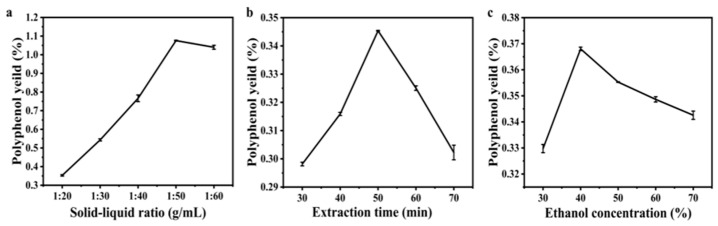
Effects of different factors on ultrasonic extraction of polyphenol from *H. erinaceus*. (**a**). Effect of solid–liquid ratio on polyphenol yield; (**b**). Effect of extraction time on polyphenol yield; (**c**). Effect of ethanol concentration on polyphenol yield.

The relationship between the concentration of gallic acid (x) and absorbance (y) fitted the standard curve: y = 3.8469x + 0.0075. Polyphenol content was calculated based on this regression. The polyphenol content initially increased, then decreased with a rising solid–liquid ratio. The highest polyphenol content was achieved at a solid–liquid ratio of 1:50, after which it continuously declined with further increasing solid–liquid ratio (Figure 1a). This may be attributed to the fact that a low solid–liquid ratio restricts sufficient contact between the sample and the solvent, while an excessively high ratio may increase the dissolution of impurities and even cause the degradation of polyphenols [24]. Extraction time significantly impacted polyphenol dissolution, reaching its peak at 50 min. At this point, polyphenol was mostly dissolved, and extending the ultrasonic time beyond 50 min resulted in decreased polyphenol content (Figure 1b). This may be attributed to the loss of target bioactive components caused by excessive extraction. The effect of ethanol concentration on polyphenol extraction yield follows a similar pattern, peaking at 40% ethanol concentration. Beyond this point, the polyphenol content decreases consistently (Figure 1c). As documented by Rahimah et al., 40% ethanol offers the optimal polarity for recovering phenolic compounds from edible mushrooms, as higher ethanol levels increase the solubility of unwanted macromolecules and reduce the extraction selectivity for polyphenols [25].

#### 2.1.2. Polyphenol BBD Analysis

The extraction temperature, extraction time and solid–liquid ratio were further optimized by BBD based on the results of single-way experiments. Table 1 shows the experimental data of BBD under different combinations of extraction conditions. Table 2 shows the results of ANOVA. Accordingly, the second-order polynomial equation was established as follows:

$$Y=-9.16012+0.123691\times A+0.143075\times B+0.162185\times C-\;0.0001375\times A\times B-7.5\;\times \;10^{-5}\times A\times C+3\;\times \;10^{-5}\times B\times C-\;0.0014058\times A^{2}-0.0013893\times B^{2}-0.0015433\times C^{2}$$

From Table 2, it is evident that the model’s regression is highly significant (*p* < 0.0001), with a lack of fit of 0.3369, exceeding the significance threshold of 0.05. This indicates a strong model fit, capable of depicting the relationship between the response value and various factors. ANOVA showed that the model was significant, while the linear term of extraction time (A) was insignificant (*p* = 0.7567). Primary items B, along with secondary factors A^2^, B^2^ and C^2^, exhibited extremely significant differences. The effects of the three factors on polyphenol extraction ranked as follows: Solid–liquid ratio (B) > Ethanol concentration (C) > Extraction time (A). This ranking was observed despite the insignificance of the linear term for A, which could be attributed to the highly significant quadratic term (A^2^, *p* < 0.0001) masking the non-significance of its linear term.

#### 2.1.3. Interactions in BBD Experiments of Polyphenol Extraction

Based on the fitted equations, three-dimensional response surface plots of different factors on the composite scores were developed using Design-Expert 8.0.6 software.

**Figure 2:**
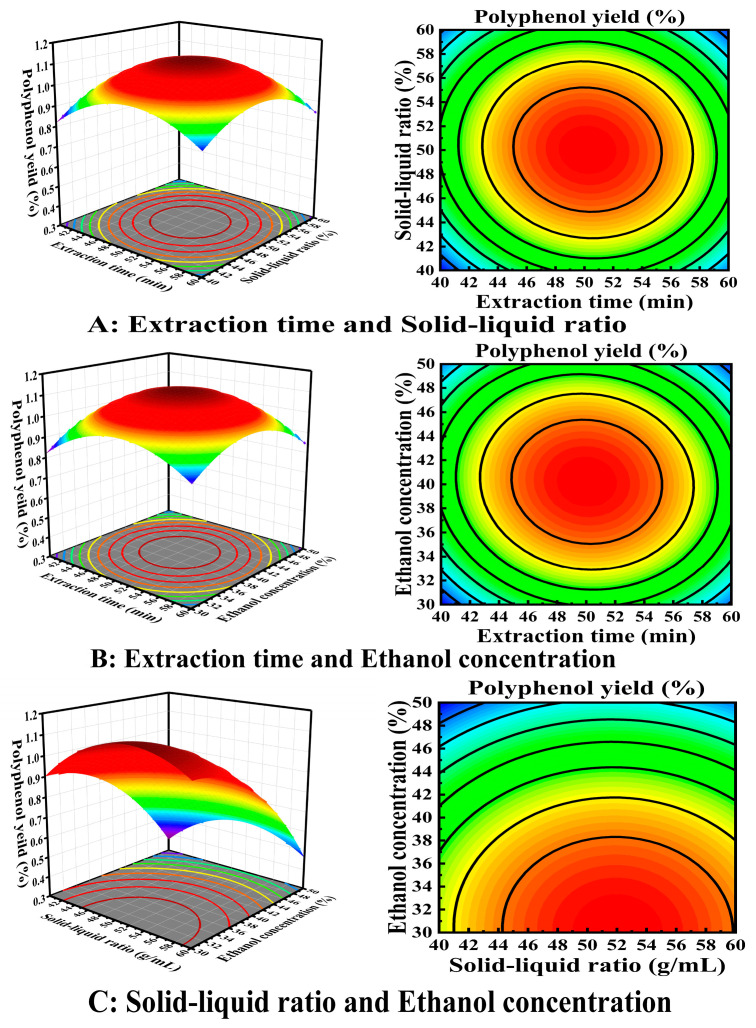
Response Surface and contour map of interaction between three factors affecting *H. erinaceus* polyphenol extraction content.

As shown in Figure 2, the interaction between the polyphenol extraction variables was visualized on a three-dimensional surface reflecting the combined effect of a number of factors on the response values when they intersect. Figure 2A shows that when the ethanol concentration remained constant, the yield first increased with increasing extraction time. After the highest yield was reached, the yield decreased with increasing extraction time; the yield increased and then decreased as the solid–liquid ratio increased. Figure 2B shows that when the solid–liquid ratio was constant, the polyphenol yield first showed a rapid upward trend with increasing extraction time. When the extraction time increased to a certain value, the polyphenol yield decreased immediately; the polyphenol yield decreased with increasing ethanol concentration. Figure 2C shows that when the extraction time was constant, the polyphenol yield first increased and then decreased with increasing solid–liquid ratio and the ethanol concentration increased; the polyphenol yield first increased, but after the highest peak was reached, the yield decreased with the solid–liquid ratio. The results show that the response surface plot for AC is steeper for this interaction term, indicating that AC has a significant effect on the composite score.

#### 2.1.4. Experimental Verification of Polyphenol Extraction

The optimal extraction conditions of polyphenol from *H. erinaceus* were obtained by the Box–Behnken experiment, under the extraction conditions of extraction time 56.85 min, solid–liquid ratio 1:56.71 g/mL, and ethanol concentration 44.64%; the theoretical prediction value of polyphenol yield was 0.9960%. We repeated the experiment three times under those conditions, and the average polyphenol yield from *H. erinaceus* was 0.9985 ± 0.03%, which is basically consistent with the predicted value and further verifies the feasibility of the theoretical model. Notably, the targeted components in this extraction study mainly comprised polyphenolic compounds (such as protocatechuic acid, gallic acid, p-coumaric acid) from *H. erinaceus*, and the optimized conditions were precisely screened to enable efficient extraction of these bioactive substances.

### 2.2. Analysis of the Extracts by LC-MS

**Figure 3:**
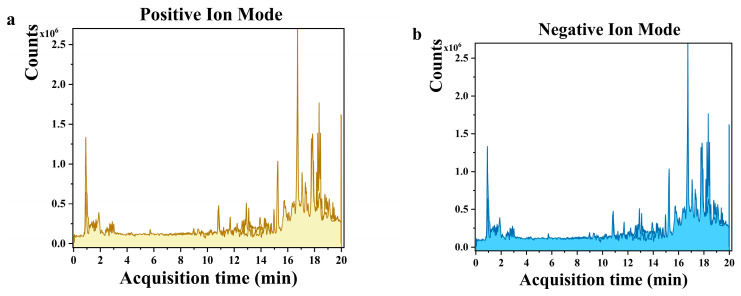
Positive and negative ion mode spectra. (**a**) Positive ion mode spectra; (**b**) negative ion mode spectra.

The crude extract of *H. erinaceus* was analyzed using LC-MS with electrospray ionization. Figure 3 shows the spectra for both positive and negative ion modes.

By comparing the acquired high-resolution mass spectrometry data against an established spectral library, 48 and 64 chemical constituents in negative ion modes (Table 3) and positive ion modes (Table 4) were tentatively identified, respectively. These constituents cover multiple compound classes, including primary metabolites such as sugar alcohols, organic acids, free amino acids, and small peptides, as well as lipid components including glycerophospholipids, fatty acids and their oxidized derivatives, together with several terpenoids, steroids and mushroom-specific bioactive components. In addition, compounds including D-arabitol, D-(+)-malic acid, and uridine diphosphate-N-acetylglucosamine are consistent with the results reported by Sevindik [11]. The results confirm the presence of multiple bioactive compounds in the extract, though their specific biological functions remain to be further validated experimentally.

### 2.3. Antioxidant Activity

**Figure 4:**
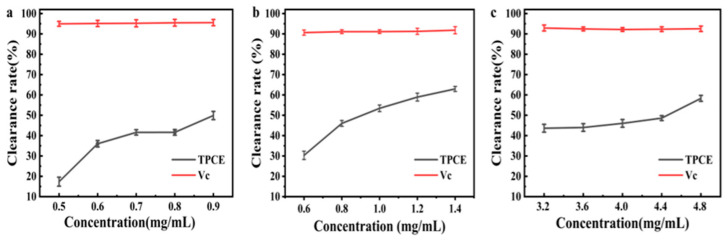
The scavenging activities of TPCE against three types of free radicals. (**a**) ABTS^+^·, (**b**) ·OH, (**c**) DPPH·.

As depicted in Figure 4, the scavenging effects of TPCE on ABTS^+^·, ·OH, and DPPH· radicals show a concentration-dependent enhancement with increasing TPCE concentration. TPCE showed markedly different scavenging capacities toward the three free radicals. Although its scavenging activity was lower than that of Vc within the tested concentration range, TPCE is a complex mixture rather than a monomeric compound, indicating its potential for strong antioxidant activity. Specifically, *EC*_50_ values of TPCE for ABTS^+^·, ·OH, and DPPH· were calculated as 0.8850 mg/mL, 0.9490 mg/mL, and 4.198 mg/mL, respectively. Compared with the reported *EC*_50_ values for analogous natural and edible mushroom extracts in the literature, the *EC*_50_ values determined in the present study fall well within the range of crude extracts with moderate antioxidant activity, except for a noticeably weaker DPPH radical scavenging capacity [26,27]. Based on LC-MS profiling, primary metabolites including D-arabitol, D-(+)-malic acid, uridine diphosphate-N-acetylglucosamine, together with terpenoids and mushroom-specific bioactive compounds, were suggested to contribute collectively to the antioxidant effects observed in this study, which was reported in the findings of Sevindik, M. et al. [11].

### 2.4. Enzyme Inhibitory Activity

**Figure 5:**
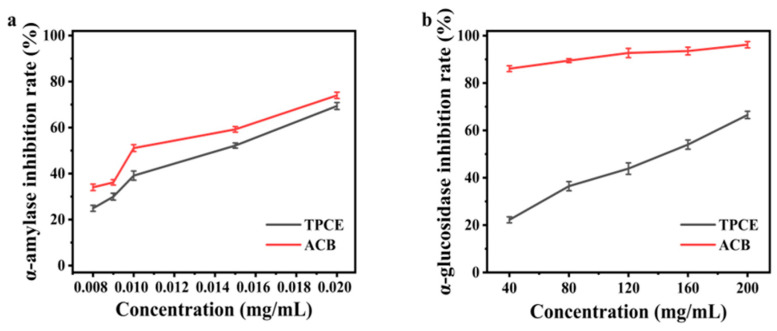
Inhibition of two enzymatic activities by different concentrations of TPCE. (**a**) α-amylase, (**b**) α-glucosidase.

As shown in Figure 5a, the inhibition of α-amylase by TPCE intensified with increased concentrations. The inhibition rate improved from 24.91 ± 1.31% to 69.36 ± 1.49% as TPCE concentrations increased from 0.008 to 0.020 mg/mL, with an *IC*_50_ determined at 0.0135 mg/mL. Then, the inhibition of α-glucosidase by TPCE intensified with increased concentrations, as depicted in Figure 5b. The inhibition rate improved from 22.22 ± 1.26 to 66.54 ± 1.53% as TPCE concentrations increased from 40 to 200 mg/mL, with an *IC*_50_ determined at 130.3 mg/mL. For the reaction of the positive control, the *IC*_50_ values of ACB on the α-amylase and α-glucosidase enzymes were 0.0113 mg/mL and 2.028 mg/mL, respectively.

The results demonstrated that TPCE exhibited favorable inhibitory activity against α-amylase, with *IC*_50_ data similar to that of the positive control drug. However, its inhibitory effect on α-glucosidase was weaker than that of the positive control. When compared with literature data for similar wild edible mushroom crude extracts, the *IC*_50_ values of TPCE against both α-amylase and α-glucosidase fell within the reported ranges for mushroom-derived hypoglycemic agents. Regarding the enzyme inhibitory activity, multiple compounds identified in TPCE and widely reported in the literature exerted potent α-amylase and α-glucosidase inhibitory effects, including polysaccharides, phenolic derivatives, terpenoids, malic acid, and sugar alcohols, which are believed to act synergistically to contribute to the observed antidiabetic potential of TPCE [28,29]. TPCE, as a crude extract, exhibited *IC*_50_ against α-amylase very close to that of the positive control ACB, indicating its potent inhibitory activity against this enzyme. Given that TPCE is a crude extract while the positive control is a pure compound, these findings indicate that TPCE possesses potential for development as an antidiabetic agent. In future studies, we will further investigate the active monomers present in TPCE.

## 3. Materials and Methods

### 3.1. Reagents and Materials

Materials: Dried *H. erinaceus* from Jilin Province was crushed and sieved through an 80 mesh sieve. Folin–Ciocalteu reagent (10% (*v*/*v*)) and anhydrous ethanol analytical grade) were acquired from Beijing Chemical Reagent Co., Ltd. (Beijing, China). Gallic acid standard, L-ascorbic acid (Vc), ferrous sulfate, 2,2-Diphenyl-1-Picrylhydrazyl (DPPH), hydrogen peroxide, salicylic acid, 2,2′-Azinobis-(3-Ethylbenzthiazoline-6-Sulphonic acid) (ABTS), Acarbose (ACB), α-glucosidase, and α-amylase were purchased from Shanghai Yuanye Biotechnology Co., Ltd. (Shanghai, China). DNS reagent came from Beijing Soleberg Technology Co., Ltd. (Beijing, China).

Equipment: Numerical control ultrasonic cleaner (Kunshan Hechuang Ultrasonic Instrument Co., Ltd., Kunshan, China); Rotary evaporator (Heidolph, Schwabach, Germany); High-speed refrigerated centrifuge (Sigma, Cream Ridge, NJ, USA); Multifunctional microplate reader (Molecular Devices, San Jose, CA, USA); UHPLC-QTOF system (Agilent Technologies Co., Ltd., Santa Clara, CA, USA).

### 3.2. Optimization of Polyphenol Extraction Process

#### 3.2.1. Polyphenol Standard Curve

Polyphenol content was quantified using a standard curve with gallic acid as the reference standard, the most widely adopted compound for Folin–Ciocalteu (F-C) total phenolic analysis due to its stability, consistent reactivity with F-C reagent, and common use in phytochemical studies [29]. Concentrations of 0.016–0.08 mg/L gallic acid standard solution were added. Another 0.5 mL of 10% F-C reagent and 1 mL of 10% Na_2_CO_3_ were added in sequence. Then, the solution was placed in the dark for 0.5 h, and the absorbance (A) was measured at a wavelength of 760 nm. The abscissa was taken as the solution concentration, and the ordinate was taken as the absorbance A_760_ to form a standard working curve [30].

#### 3.2.2. Determination of Polyphenol Yield

Following extraction, the polyphenol yield from *H. erinaceus* was calculated according to the following formula [31]:

(1) Y=(C×V×n/m)×100%

where C is the mass concentration of *H. erinaceus* polyphenol calculated according to the absorption value, %; V is the total amount of *H. erinaceus* polyphenol extract volume, mL; m is the weight of *H. erinaceus*, mg; and n is the dilution ratio.

#### 3.2.3. Effect of Solid–Liquid Ratio on Polyphenol Yield

Five parts of 1 g of *H. erinaceus* power were accurately weighed in a 100 mL Erlenmeyer flask, the extraction conditions were fixed, with an ethanol solvent of 60%, extraction time of 50 min, ultrasonic power of 400 W, and extraction temperature of 25 °C. Subsequently, a single-factor test was conducted by adjusting the solid–liquid ratio. Each experiment was duplicated twice, and the two extracts were merged. The filtrate was concentrated under reduced pressure and djusted to 5 mL with 60% ethanol prior to the determination of polyphenol yield.

#### 3.2.4. Effect of Extraction Time on Polyphenol Yield

After accurately weighing five aliquots of 1 g each of *H. erinaceus* power and transferring each aliquot to a 100 mL Erlenmeyer flask, the extraction conditions were kept constant: 60% ethanol was used as the extraction solvent, the solid–liquid ratio was set at 1:20 (g/mL), the ultrasonic power was fixed at 400 W, and the temperature was maintained at 25 °C. Subsequently, a single-factor experiment was performed by varying only the extraction time. Each experiment was duplicated twice, and the two extracts were merged. The filtrate was concentrated under reduced pressure, and djusted to 5 mL with 60% ethanol, prior to the determination of polyphenol yield.

#### 3.2.5. Effect of Ethanol Concentration on Polyphenol Yield

Five 1 g aliquots of *H. erinaceus* power were precisely weighed, with each aliquot placed into a 100 mL Erlenmeyer flask; the extraction parameters were maintained at constant values: a solid–liquid ratio of 1:20 (g/mL), extraction duration of 50 min, ultrasonic power of 400 W, and ultrasonic temperature of 25 °C. A single-factor assay was then performed, where the only variable was the concentration of ethanol used for extraction. Each experiment was duplicated twice, and the two extracts were merged. The filtrate was concentrated under reduced pressure and djusted to 5 mL with 60% ethanol prior to the determination of polyphenol yield.

#### 3.2.6. Polyphenol Extraction BBD Response Surface Method Experimental Design

The experimental scheme was obtained using Design-Expert 8.0 software and BBD. Based on the results of the one-factor test, the main factors with significant effects on polyphenol yield were selected, and according to the principle of Box–Behnken central combination experimental design, a three-factor, three-level central composite design was carried out with the polyphenol yield of *H. erinaceus* as the response value. The experiment was repeated three times, and the polyphenol yield response values were obtained and averaged for data analysis. As shown in Table 5, the experimental factors and levels used the fitted second-order polynomial model volume:

$$Y=\sum_{i=1}^{3}\beta_{i}X_{i}+\sum_{i=1}^{3}\beta_{ii}X_{i}^{2}+\sum_{i=1}^{2}\sum_{j=i+1}^{3}\beta_{ij}X_{i}X_{j}$$

where Y is the predicted response; β_i_, β_ii_ and β_ij_ are regression coefficients of intercept term, linear term, quadratic term and interaction term, respectively, and X_i_ and X_j_ are coding parameters.

### 3.3. LC-MS Analysis

LC-MS analysis was performed using an Agilent 1290II-6545 UHPLC-QTOF system, equipped with a Waters (Milford, MA, USA) ACQUITY UPLC BEH C_18_ (2.1 × 50 mm, 1.7 µm). The mobile phase consisted of two components; mobile phase A comprised 0.1% formic acid in water, while mobile phase B was 0.1% formic acid solution in acetonitrile. Data were collected in both positive and negative ionization modes. The full scan was conducted at 100 to 3000 *m*/*z*.

*H. erinaceus* extract was prepared using 80% methanol solution, filtered with a 0.22 µm microporous nylon membrane, and then evaluated.

### 3.4. Determination of Antioxidant Activity

#### 3.4.1. ABTS^+^· Radical Scavenging Assay

An ABTS^+^· radical scavenging capacity assay was performed with minor modifications in reference to the method of Re et al. [32]. To evaluate the ABTS^+^· radical scavenging capacity of TPCE, a reaction mixture consisting of 7 mM ABTS reagent and 140 mM potassium persulfate (K_2_S_2_O_8_) was prepared. This mixture was then incubated in the dark at 4 °C for 12 h, to facilitate the generation of ABTS^+^· radicals. Subsequently, 100 μL of TPCE samples with various concentrations were separately mixed thoroughly with 100 μL of the ABTS^+^· stock solution. After standing in the dark for 30 min, the absorbance of each mixture was measured at a wavelength of 734 nm. Each group of experiments was performed in triplicate to ensure the reliability and reproducibility of the experimental results. The absorbance of the sample reaction system was denoted as A_i_, while A_0_ represented the absorbance measured by substituting the sample with the sample solvent. A_j_ was defined as the absorbance obtained when the ABTS^+^· solution was replaced by the sample solvent. Vc served as the positive control, and the ABTS^+^· radical scavenging activity was calculated by applying the following formula:

(2) $${\mathrm{ABTS}}^{+.}\;\mathrm{radical}\;\mathrm{scavenging}\;\mathrm{rate}\;=\;\left(1\;-\;(A_{i}\;-\;A_{j})/A_{0}\right)\;\times \;100\%$$

#### 3.4.2. OH· Radical Scavenging Assay

The free radical was determined using the method of Smirnof and Geng with minor modifications [33,34]. A volume of 50 μL TPCE sample solutions was mixed with 50 μL of 50 mmol/L ferrous sulfate (FeSO_4_) solution, 50 μL of 10 mmol/L salicylic acid–ethanol solution, and 50 μL of 3% hydrogen peroxide (H_2_O_2_) solution. The resulting mixture was incubated in the dark for 30 min. Triplicate measurements were carried out for each experimental group to ensure the reliability and repeatability of the results. After the incubation period, the absorbance of reaction system (designated as A_reaction_) was measured at a wavelength of 510 nm. For the control group (A_control_), distilled water was used to replace the salicylic acid–ethanol solution, and the corresponding absorbance was recorded. For the blank group (A_blank_), the sample was substituted with the sample solvent, and its absorbance was measured as well. Vc was employed as the positive control, and the OH· radical scavenging capacity was computed in accordance with the following formula:

(3) $${OH}^{.}\;radical\;scavenging\;rate=\left(1-\frac{A_{reaction}-A_{control}}{A_{blank}}\right)\times 100\%$$

where $A_{reaction}$ represents the absorbance of the sample, $A_{control}$ represents the absorbance of the blank control group, and $A_{blank}$ represents the absorbance of the sample without hydrogen peroxide.

#### 3.4.3. DPPH· Radical Scavenging Assay

The experiments were carried out using the method described in Brand-Williams [35]. TPCE samples with different concentration gradients (100 μL each) were individually mixed with 100 μL of DPPH· solution. Each experimental group was run in triplicate to ensure that the experimental results were reliable and reproducible. After incubating the mixture for 30 min, the absorbance (denoted as A_i_) was determined at a wavelength of 517 nm. The control absorbance values were recorded: A_j_ was measured by substituting the TPCE sample with the sample solvent, while A_c_ was obtained by replacing the DPPH· solution with anhydrous ethanol. Vc was used as the positive control, and the DPPH· radical scavenging capacity was calculated by means of the following formula:

(4) $${DPPH}^{.}\;clearance=\left(1-(A_{i}-A_{j})/A_{c}\right)\times 100\%$$

### 3.5. Determination of Inhibition Rate

#### 3.5.1. Determination of α-Amylase Inhibition Rate

The experiments were carried out using the method described in Padilla-Camberos, et al. [36]. TPCE solutions (0.008–0.020 mg/mL) in volumes of 100 µL were mixed with 50 µL of α-amylase (1.0 U/mL); following a 15 min incubation period at 37 °C, 50 µL of 1% (*w*/*v*) soluble starch was added, and the solutions were then incubated for a further 10 min. Subsequently, 100 µL of DNS was introduced into the mixture, and then heated at 100 °C for 5 min. After cooling, 1 mL of distilled water was added, and the absorbance at 540 nm was measured. Each experimental group was run in triplicate to ensure that the experimental results were reliable and reproducible. The inhibition rate was calculated using the following equation:

(5) $$Inhibition\;rate=\left(1-(A_{1}-A_{2})/(A_{0}-{A_{0}}^{\prime })\right)\times 100\%$$

where A_1_, A_2_, A_0_, and A_0_ are the absorbances at 540 nm for the sample group, blank sample group, control group, and blank control group, respectively. *IC*_50_ was determined from the regression equation of the inhibition curve.

#### 3.5.2. Determination of α-Glucosidase Inhibition Rate

The experiments were carried out using the method described in Yao et al. [37]. TPCE solutions (5–160 mg/mL) in volumes of 100 µL were mixed with 50 µL of α-glucosidase solution (0.5 U/mL). The mixture was subsequently incubated at 37 °C for 15 min. Subsequently, 100 µL of PNPG (5.0 mmol/L) was added to the reaction mixture, which was then incubated at 37 °C for 10 min. The reaction was terminated by the addition of 750 µL of Na_2_CO_3_ (1.0 mol/L), and the absorbance was recorded at 405 nm. Triplicate measurements were carried out for each experimental group to ensure the reliability and repeatability of the results. The inhibition rate was calculated using the following equation:

(6) $$Inhibition\;rate=\left(1-(A_{1}-A_{2})/(A_{0}-{A_{0}}^{\prime })\right)\times 100\%$$

where A_1_, A_2_, A_0_, and A_0_ are the absorbances at 405 nm for the sample group, blank sample group, control group, and blank control group, respectively. *IC*_50_ was determined from the regression equation of the inhibition curve.

## 4. Conclusions

In this research, ultrasound-assisted extraction was employed to extract polyphenols from *H. erinaceus*, and the effects of extraction duration, solid–liquid ratio, and ethanol concentration on polyphenol yield were systematically evaluated. Subsequent optimization via BBD response surface methodology determined the optimal extraction parameters: extraction time of 56.85 min, solid–liquid ratio of 1:56.71 g/mL, and ethanol concentration of 44.64%, under which the TPCE yield reached 0.9985 ± 0.03%, verifying the effectiveness and reproducibility of the optimized extraction protocol for *H. erinaceus* polyphenols. In addition, LC-MS analysis tentatively identified multiple bioactive constituents in TPCE (including polysaccharides, phenolics, terpenoids, malic acid, and sugar alcohols), laying a preliminary chemical foundation for the observed bioactivities. In vitro bioactivity assessments revealed that TPCE possessed notable free radical scavenging capacity, with the potency ranking ABTS^+^· > ·OH > DPPH·, corresponding to *EC*_50_ values of 0.8850 mg/mL, 0.9490 mg/mL, and 4.198 mg/mL, respectively. Furthermore, TPCE exhibited remarkable inhibitory activity against carbohydrate-digesting enzymes, with *IC*_50_ values of 0.0135 mg/mL for α-amylase and 130.3 mg/mL for α-glucosidase; the *IC*_50_ of ACB on α-amylase was 0.0113 mg/mL, indicating that TPCE possesses favorable inhibitory activity against α-amylase. Collectively, these quantitative and qualitative findings provide robust scientific evidence supporting the nutritional and medicinal value of *H. erinaceus* and highlight its promising application potential as a natural adjudicative agent for blood glucose regulation and oxidative stress relief.

## Figures and Tables

**Table 1:** BBD experimental results.

Number	A: Extraction Time (min)	B: Solid–Liquid Ratio (g/mL)	C: Ethanol Concentration (%)	Polyphenol Yield (%)
1	60	50	30	0.8399
2	40	40	40	0.7616
3	50	50	40	1.1139
4	50	40	30	0.7458
5	50	60	30	0.8965
6	50	40	50	0.7682
7	60	50	50	0.8291
8	40	60	40	0.8741
9	50	50	40	1.1038
10	40	50	30	0.8239
11	40	50	50	0.8681
12	60	40	40	0.7729
13	50	50	40	1.1309
14	60	60	40	0.8974
15	50	50	40	1.1305
16	50	60	50	0.8889
17	50	50	40	1.1197

**Table 2:** Regression equation model and analysis of variance.

Source	Sum of Squares	df	Mean Square	F-Value	*p*-Value
Model	0.3294	9	0.0366	225.88	<0.0001 ***
A	0	1	0	0.1038	0.7567
B	0.0323	1	0.0323	199.37	<0.0001 ***
C	0.0003	1	0.0003	1.79	0.2225
AB	0	1	0	0.2221	0.6517
AC	0.0008	1	0.0008	4.67	0.0676
BC	0.0002	1	0.0002	1.39	0.2772
A^2^	0.0813	1	0.0813	501.49	<0.0001 ***
B^2^	0.1003	1	0.1003	618.83	<0.0001 ***
C^2^	0.0832	1	0.0832	513.47	<0.0001 ***
Residual	0.0011	7	0.0002		
Lack of Fit	0.0006	3	0.0002	1.53	0.3369
Pure Error	0.0005	4	0.0001		
Cor Total	0.3306	16			
				R^2^	0.9966
				R^2^ Adj	0.9922
				R^2^ Pre	0.9682
				Adeq Precision	38.0992

Note: *** indicates extremely significant differences, *p* < 0.001.

**Table 3:** Results of the negative ion modes.

NO.	Retention Time (min)	Mass-to-Charge Ratio (*m*/*z*)	Name	Formula
1	0.877	227.0775	L-Iditol	C_6_H_14_O_6_
2	0.903	487.1816	Ala His Met Met	C_19_H_32_N_6_O_5_S_2_
3	0.908	181.0728	Lathyrine	C_7_H_10_N_4_O_2_
4	0.922	573.1516	Cys Asp Glu Tyr	C_21_H_28_N_4_O_10_S
5	0.932	355.0936	5-Hydroxy-4-methoxy-3-methyl-2,6-canthinedione	C_16_H_12_N_2_O_4_
6	0.934	231.0311	9-Hydroxy-4-methoxypsoralen	C_12_H_8_O_5_
7	0.94	387.1175	Fructoselysine 6-phosphate	C_12_H_25_N_2_O_10_P
8	0.945	311.1028	Flurprimidol	C_15_H_15_F_3_N_2_O_2_
9	0.966	439.0866	3,5-Dihydroxyphenyl 1-O-(6-O-galloyl-beta-D-glucopyranoside)	C_19_H_20_O_12_
10	0.97	151.062	D-Arabitol	C_5_H_12_O_5_
11	1.017	133.0154	D-(+)-Malic acid	C_4_H_6_O_5_
12	1.026	267.0742	cis-ACCP	C_7_H_15_N_2_O_4_P
13	1.065	606.0768	Uridine diphosphate-N-acetylglucosamine	C_17_H_27_N_3_O_17_P_2_
14	1.074	191.021	Citric acid	C_6_H_8_O_7_
15	1.345	191.0201	2,3-Dioxogulonic acid	C_6_H_8_O_7_
16	1.477	231.0159	6-Cyano-7-nitroquinoxaline-2,3-dione	C_9_H_4_N_4_O_4_
17	1.673	115.0039	Formylpyruvate	C_4_H_4_O_4_
18	10.804	740.4941	PS(P-16:0/18:3(9Z,12Z,15Z))	C_40_H_72_NO_9_P
19	12.616	429.1565	Thr His Cys Ala	C_16_H_26_N_6_O_6_S
20	12.863	429.1562	17β-hydroxy Wortmannin	C_23_H_26_O_8_
21	13.911	331.156	Gibberellin A20	C_19_H_24_O_5_
22	14.731	415.1771	Erioflorin methacrylate	C_23_H_28_O_7_
23	15.716	293.212	alpha-kamlolenic acid	C_18_H_30_O_3_
24	15.733	295.2283	9(R)-HODE	C_18_H_32_O_3_
25	15.982	265.1486	Lauryl hydrogen sulfate	C_12_H_26_O_4_S
26	16.378	564.3322	PE(20:2(11Z,14Z)/0:0)	C_25_H_48_NO_7_P
27	16.547	459.2976	Lys Lys Ser Val	C_20_H_40_N_6_O_6_
28	16.615	489.3084	Phe Ile Ile Val	C_26_H_42_N_4_O_5_
29	16.724	333.2295	9,10-dihydroxy-hexadecanoic acid	C_16_H_32_O_4_
30	16.74	379.2376	Emopamil	C_23_H_30_N_2_
31	16.764	279.2338	9(E),11(E)-Conjugated Linoleic Acid	C_18_H_32_O_2_
32	16.787	459.3003	Eicosapentaenoyl Serotonin	C_30_H_40_N_2_O_2_
33	16.813	311.1691	N-Undecylbenzenesulfonic acid	C_17_H_28_O_3_S
34	16.936	339.2334	1,1′-[1,11-Undecanediylbis(oxy)]bisbenzene	C_23_H_32_O_2_
35	17.116	435.2983	Termitomycamide B	C_28_H_40_N_2_O_2_
36	17.31	669.5314	Glycerol 1-dodecanoate 2-tetradecanoate 3-octanoate	C_37_H_70_O_6_
37	17.317	281.2494	C18:1n-13	C_18_H_34_O_2_
38	17.372	451.2842	PA(19:0/0:0)	C_22_H_45_O_7_P
39	17.407	461.3134	Sorbitan palmitate	C_22_H_42_O_6_
40	17.581	325.1848	4-Dodecylbenzenesulfonic acid	C_18_H_30_O_3_S
41	17.766	279.235	Linoleic acid	C_18_H_32_O_2_
42	17.793	413.1007	Dihydrogriseofulvin	C_17_H_19_C_l_O_6_
43	17.845	293.1802	Sodium Tetradecyl Sulfate	C_14_H_30_O_4_S
44	17.926	407.2488	Dimethamine	C_24_H_32_N_4_O_2_
45	18.393	323.2209	TOFA	C_19_H_32_O_4_
46	18.408	255.2335	Palmitic Acid	C_16_H_32_O_2_
47	18.786	415.1156	BAY-u3405	C_21_H_21_FN_2_O_4_S
48	19.453	319.2321	20-HETE	C_20_H_32_O_3_

**Table 4:** Results of the positive ion modes.

NO.	Retention Time (min)	Mass-to-Charge Ratio (*m*/*z*)	Name	Formula
1	0.86	104.106	Choline	C_5_H_14_NO
2	0.971	148.0601	L-Glutamic acid	C_5_H_9_NO_4_
3	1.01	258.1098	Glycerophosphocholine	C_8_H_21_NO_6_P
4	1.064	118.0855	L-Valine	C_5_H_11_NO_2_
5	1.689	129.0543	Methyl 4-oxo-2-pentenoate	C_6_H_8_O_3_
6	1.867	132.1024	L-Leucine	C_6_H_13_NO_2_
7	2.739	166.0851	DL-Phenylalanine	C_9_H_11_NO_2_
8	10.841	701.4922	PC(10:0/18:0)	C_36_H_73_NO_8_P
9	13.05	353.1347	Gibberellin A95	C_19_H_22_O_5_
10	13.943	355.1492	Dinocton	C_16_H_22_N_2_O_7_
11	15.053	365.1321	Ser Ser Gly Asp	C_12_H_20_N_4_O_9_
12	15.17	301.1393	7-Methylinosine	C_11_H_15_N_4_O_5_
13	15.225	579.286	Ala Arg Asp Pro Val	C_23_H_40_N_8_O_8_
14	15.281	279.1563	Monomenthyl succinate	C_14_H_24_O_4_
15	15.629	357.2574	8-iso Prostaglandin E2-d4	C_20_H_28_D_4_O_5_
16	15.668	401.2831	Arg Ile Ile	C_18_H_36_N_6_O_4_
17	15.723	445.3087	17-phenyl trinor Prostaglandin F2α cyclopropyl amide	C_26_H_37_NO_4_
18	15.846	489.3342	(17Z)-1α,25-dihydroxy-26,27-dimethyl-17,20,22,22,23,23-hexadehydro-24a,24b-dihomovitamin D3/(17Z)-1α,25-dihydroxy-26,27-dimethyl-17,20,22,22,23,23-hexadehydro-24a,24b-dihomocholecalciferol	C_31_H_46_O_3_
19	16.371	520.3338	Thr Arg Arg Ala	C_19_H_38_N_10_O_6_
20	16.384	542.3155	Asn Pro Arg Arg	C_21_H_39_N_11_O_6_
21	16.393	639.3999	PA(18:2(9Z,12Z)/12:0)	C_33_H_61_O_8_P
22	16.435	595.3745	PS(22:2(13Z,16Z)/0:0)	C_28_H_52_NO_9_P
23	16.553	437.2822	*Hericerin*	C_27_H_33_O_3_
24	16.592	385.2883	Kalkitoxin thioamide alcohol	C_21_H_40_N_2_O_2_S
25	16.598	399.2676	10′-apo-beta-carotenal	C_27_H_36_O
26	16.615	473.3394	Phylloquinone	C_31_H_46_O_2_
27	16.709	341.2626	12-epi Leukotriene B4-d4	C_20_H_28_D_4_O_4_
28	16.712	429.3139	Fesoterodine	C_26_H_37_NO_3_
29	16.722	781.5185	PI(P-18:0/13:0)	C_40_H_77_O_12_P
30	16.734	357.2218	(R,E)-S-2-acetamido-13-(methylamino)-13-oxotridec-3-enyl ethanethioate	C_18_H_32_N_2_O_3_S
31	16.753	693.4676	PG(12:0/18:1(9Z))	C_36_H_69_O_10_P
32	16.766	605.4164	PA(17:1(9Z)/12:0)	C_32_H_61_O_8_P
33	16.775	737.4934	PC(18:3(9Z,12Z,15Z)/13:0)	C_39_H_73_NO_8_P
34	16.792	437.2832	Pangamic acid	C_20_H_40_N_2_O_8_
35	16.866	425.2824	Lys Lys Lys	C_18_H_38_N_6_O_4_
36	16.874	413.2824	1α-fluoro-25-hydroxy-16,17,23,23,24,24-hexadehydrovitamin D3/1α-fluoro-25-hydroxy-16,17,23,23,24,24-hexadehydrocholecalciferol	C_27_H_37_FO_2_
37	16.888	522.3488	Dipyridamole	C_24_H_40_N_8_O_4_
38	17.102	439.2978	Apo-8′-lycopenal	C_30_H_40_O
39	17.501	629.4884	1-(6-[5]-ladderane-hexanyl)-2-(8-[3]-ladderane-octanyl)-sn-glycerol	C_41_H_66_O_3_
40	17.646	457.3445	N-arachidonoyl vanillylamine	C_28_H_41_NO_3_
41	17.665	427.2985	Leupeptin	C_20_H_38_N_6_O_4_
42	17.667	501.3704	Deterrol stearate	C_33_H_50_O_2_
43	17.689	589.422	Arg Arg Leu Lys	C_24_H_49_N_11_O_5_
44	17.788	809.5503	PI(P-20:0/13:0)	C_42_H_81_O_12_P
45	17.797	853.5748	PS(22:6(4Z,7Z,10Z,13Z,16Z,19Z)/18:0)	C_46_H_78_NO_10_P
46	17.798	721.4987	PG(14:0/18:1(9Z))	C_38_H_73_O_10_P
47	17.804	585.4637	3,4-Dihydrospheroidenone	C_41_H_60_O_2_
48	17.804	765.5238	Spinetoram	C_42_H_69_NO_10_
49	17.832	435.3627	Petrosterol	C_29_H_48_O
50	17.835	479.3884	2-Tricosanamidoethanesulfonic acid	C_25_H_51_NO_4_S
51	17.838	677.4725	Lansioside A	C_38_H_61_NO_8_
52	17.864	633.447	PA(17:0/14:1(9Z))	C_34_H_65_O_8_P
53	17.867	369.2934	2,3-dinor-6-keto Prostaglandin F1α-d9	C_18_H_21_D_9_O_6_
54	17.875	545.396	Daphniphylline	C_32_H_49_NO_5_
55	17.918	413.3194	N-Octadecyl-N’-propyl-sulfamide	C_21_H_46_N_2_O_2_S
56	17.944	431.2388	Deoxypyridinoline	C_18_H_29_N_4_O_7_
57	18.129	515.3321	Ile Leu Arg Asn	C_22_H_42_N_8_O_6_
58	18.256	475.3397	5beta-Cholestane-3alpha,7alpha,12alpha,23,26-pentol	C_27_H_48_O_5_
59	18.37	441.3139	dolichyl phosphate	C_25_H_45_O_4_P
60	18.392	401.322	4,4′-Diapolycopene	C_30_H_4_0
61	19.09	381.2933	1′H-5alpha-Androst-2-eno[3,2-b]indol-17beta-ol	C_25_H_33_NO
62	19.101	803.5327	PE(22:6(4Z,7Z,10Z,13Z,16Z,19Z)/18:3(9Z,12Z,15Z))	C_45_H_72_NO_8_P
63	19.104	413.2617	Diisooctyl phthalate	C_24_H_38_O_4_
64	19.699	451.3124	N-arachidonoyl glutamic acid	C_25_H_39_NO_5_

**Table 5:** Response surface BBD experimental factors and levels.

Variables	Symbol	Coded Levels		
		−1	0	+1
Extraction time (min)	A	40	50	60
Solid–liquid ratio (g/mL)	B	40	50	60
Ethanol concentration (%)	C	30	40	50

## Data Availability

Data are contained within the article.
